# Toward Shared Decision-Making in Degenerative Cervical Myelopathy: Protocol for a Mixed Methods Study

**DOI:** 10.2196/46809

**Published:** 2023-10-09

**Authors:** Irina Sangeorzan, Grazia Antonacci, Anne Martin, Ben Grodzinski, Carl M Zipser, Rory K J Murphy, Panoraia Andriopoulou, Chad E Cook, David B Anderson, James Guest, Julio C Furlan, Mark R N Kotter, Timothy F Boerger, Iwan Sadler, Elizabeth A Roberts, Helen Wood, Christine Fraser, Michael G Fehlings, Vishal Kumar, Josephine Jung, James Milligan, Aria Nouri, Allan R Martin, Tammy Blizzard, Luiz Roberto Vialle, Lindsay Tetreault, Sukhvinder Kalsi-Ryan, Anna MacDowall, Esther Martin-Moore, Martin Burwood, Lianne Wood, Abdul Lalkhen, Manabu Ito, Nicky Wilson, Caroline Treanor, Sheila Dugan, Benjamin M Davies

**Affiliations:** 1 Myelopathy.org Cambridge United Kingdom; 2 Department of Primary Care and Public Health National Institute of Health Research (NIHR) Applied Research Collaboration (ARC) Northwest London Imperial College London London United Kingdom; 3 Centre for Health Economics and Policy Innovation (CHEPI) Business School Imperial College London London United Kingdom; 4 Faculty of Medicine, Health and Social Care Canterbury Christ Church University Canterbury United Kingdom; 5 University Hospitals Sussex NHS Foundation Trust Brighton United Kingdom; 6 Spinal Cord Injury Center Balgrist University Hospital Zurich Switzerland; 7 Department of Neurosurgery Barrow Neurological Institute St. Joseph’s Hospital and Medical Center Phoenix, AZ United States; 8 Psychology Department School of Social Sciences University of Ioannina Ioannina Greece; 9 Division of Physical Therapy School of Medicine Duke University Durham, CA United States; 10 Department of Orthopaedics School of Medicine Duke University Durham, CA United States; 11 Department of Population Health Sciences School of Medicine Duke University Durham, CA United States; 12 Duke Clinical Research Institute Duke University Durham, CA United States; 13 Sydney School of Health Sciences Faculty of Medicine and Health The University of Sydney Sydney Australia; 14 The Miami Project to Cure Paralysis The Miller School of Medicine University of Miami Miami, FL United States; 15 Division of Physical Medicine and Rehabilitation Department of Medicine University of Toronto Toronto, ON Canada; 16 The KITE Research Institute Toronto Rehabilitation Institute University Health Network Toronto, ON Canada; 17 Department of Clinical Neurosurgery University of Cambridge Cambridge United Kingdom; 18 Department of Neurosurgery Medical College of Wisconsin Milwaukee, WI United States; 19 Department of Health Sciences University of Stirling Scotland United Kingdom; 20 Physiotherapy Department National Health Service Lothian Edinburgh United Kingdom; 21 Division of Neurosurgery and Spine Program Department of Surgery University of Toronto Toronto, ON Canada; 22 Division of Neurosurgery, Krembil Neuroscience Centre Toronto Western Hospital University Health Network Toronto, ON Canada; 23 Department of Orthopaedics Postgraduate Institute of Medical Education and Research Chandigarh India; 24 Department of Orthopaedics All India Institute of Medical Sciences Deoghar India; 25 Institute of Psychiatry, Psychology & Neuroscience King's College London United Kingdom; 26 Department of Neurosurgery King's College Hospital London United Kingdom; 27 Department of Family Medicine McMaster University Hamilton, ON Canada; 28 Division of Neurosurgery Geneva University Hospitals University of Geneva Geneva Switzerland; 29 Department of Neurological Surgery University of California, Davis Davis, CA United States; 30 School of Medicine Pontifical Catholic University of Paraná Curitiba Brazil; 31 Department of Neurology New York University New York, NY United States; 32 Department of Surgery University of Toronto Toronto, ON Canada; 33 Department of Surgical Sciences Uppsala University and Department of Orthopaedics The Academic Hospital of Uppsala Uppsala Sweden; 34 Faculty of Health and Life Sciences University of Exeter Exeter United Kingdom; 35 NeuroSpinal Assessment Unit Nottingham University Hospitals NHS Trust Nottingham United Kingdom; 36 Northern Care Alliance Salford Royal NHS Foundation Trust Manchester United Kingdom; 37 Department of Orthopaedic Surgery National Hospital Organization Hokkaido Medical Center Sapporo Japan; 38 Physiotherapy Department King’s College Hospital NHS Foundation Trust London United Kingdom; 39 Department of Physiotherapy Beaumont Hospital Dublin Ireland; 40 Department of Neurosurgery Beaumont Hospital Dublin Ireland; 41 School of Physiotherapy Royal College of Surgeons in Ireland Dublin Ireland

**Keywords:** degenerative cervical myelopathy, spine, spinal cord, chronic, aging, geriatric, patient engagement, shared decision-making, process mapping, core information set, decision-making, patient education, common data element, Research Objectives and Common Data Elements for Degenerative Cervical Myelopathy, RECODE-DCM

## Abstract

**Background:**

Health care decisions are a critical determinant in the evolution of chronic illness. In shared decision-making (SDM), patients and clinicians work collaboratively to reach evidence-based health decisions that align with individual circumstances, values, and preferences. This personalized approach to clinical care likely has substantial benefits in the oversight of degenerative cervical myelopathy (DCM), a type of nontraumatic spinal cord injury. Its chronicity, heterogeneous clinical presentation, complex management, and variable disease course engenders an imperative for a patient-centric approach that accounts for each patient’s unique needs and priorities. Inadequate patient knowledge about the condition and an incomplete understanding of the critical decision points that arise during the course of care currently hinder the fruitful participation of health care providers and patients in SDM. This study protocol presents the rationale for deploying SDM for DCM and delineates the groundwork required to achieve this.

**Objective:**

The study’s primary outcome is the development of a comprehensive checklist to be implemented upon diagnosis that provides patients with essential information necessary to support their informed decision-making. This is known as a core information set (CIS). The secondary outcome is the creation of a detailed process map that provides a diagrammatic representation of the global care workflows and cognitive processes involved in DCM care. Characterizing the critical decision points along a patient’s journey will allow for an effective exploration of SDM tools for routine clinical practice to enhance patient-centered care and improve clinical outcomes.

**Methods:**

Both CISs and process maps are coproduced iteratively through a collaborative process involving the input and consensus of key stakeholders. This will be facilitated by Myelopathy.org, a global DCM charity, through its Research Objectives and Common Data Elements for Degenerative Cervical Myelopathy community. To develop the CIS, a 3-round, web-based Delphi process will be used, starting with a baseline list of information items derived from a recent scoping review of educational materials in DCM, patient interviews, and a qualitative survey of professionals. A priori criteria for achieving consensus are specified. The process map will be developed iteratively using semistructured interviews with patients and professionals and validated by key stakeholders.

**Results:**

Recruitment for the Delphi consensus study began in April 2023. The pilot-testing of process map interview participants started simultaneously, with the formulation of an initial baseline map underway.

**Conclusions:**

This protocol marks the first attempt to provide a starting point for investigating SDM in DCM. The primary work centers on developing an educational tool for use in diagnosis to enable enhanced onward decision-making. The wider objective is to aid stakeholders in developing SDM tools by identifying critical decision junctures in DCM care. Through these approaches, we aim to provide an exhaustive launchpad for formulating SDM tools in the wider DCM community.

**International Registered Report Identifier (IRRID):**

DERR1-10.2196/46809

## Introduction

### Background

Degenerative cervical myelopathy (DCM) [1,2] arises when age-related changes progressively narrow the cervical spinal cord canal, resulting in neurological dysfunction secondary to cord compression [3]. Its exact epidemiology is poorly understood, likely owing to widespread underdiagnosis [4,5]. However, estimates based on imaging studies suggest that DCM could affect as many as 1 in 50 adults [2]. Although surgical decompression can halt disease progression and afford varying levels of recovery in functional status, pain, and quality of life [6-8], most people with DCM [9] will experience lifelong residual disability despite treatment [8,10]. This is often associated with a poor quality of life [11,12], high unemployment rates [8,13], and mental health difficulties [10]. From a socioeconomic point of view, DCM places a substantial financial burden on society [14,15]. In England, United Kingdom, alone, the cumulative costs associated with disability payments, lost productivity, and hospital admission are conservatively estimated at £0.7 billion (US $0.9 billion) per annum [15]. Moreover, DCM affects not only people with DCM but also their families and caregivers, who experience a low quality of life owing to caregiving strain [16]. As the incidence of DCM is expected to increase with aging populations [17], interventions that target the human and economic cost of illness associated with this condition represent an immediate priority for global health care systems and the wider community [18]. Shared decision-making (SDM) represents one such potential cost containment and patient outcome improvement strategy that could be implemented.

### A Role for SDM in DCM

Involving patients in collaborative deliberations about their care can foster improved clinical outcomes [19,20] and curtail the needless use of health care resources [21,22]. The epitome of this patient-centric paradigm is SDM, an approach to clinical care wherein clinicians and patients work together to reach evidence-based solutions that prioritize patients’ values and preferences [23,24].

Over several decades of scholarship since its emergence in the early 1980s [25], SDM has risen to prominence as a hallmark of quality clinical practice, notably in the realm of unresolved, chronic illnesses [26]. Given the evolving and cyclic nature of many chronic conditions, continual reevaluation and adjustment of initial health management judgments are often required as patients progress through the trajectory of their illness [27-29]. Furthermore, the complexity of chronic illnesses tends to expand over time, presenting patients and health care providers with multiple options, each with its own set of benefits and drawbacks [30]. By engaging patients in the decision-making process and considering the unique circumstances and preferences of each patient along with the potential trade-offs of each option [31], SDM can facilitate the selection of the most appropriate course of action, ultimately leading to improved health outcomes [32]. Indeed, the health management decisions of people with chronic illnesses are optimal when founded on consideration of their preferences, values, and circumstances [33-36].

Therefore, the application of SDM in DCM is likely highly pertinent. People with DCM encounter care decisions that are contingent upon personal, contextual, and technical factors on a fairly recurrent basis [13,37,38]. For example, the clinical manifestation of DCM has a diverse spectrum, with symptomatology that varies from person to person [39]. In the views of people with DCM, seemingly unrelated ailments such as loss of bowel or bladder control can be traced back to the spinal cord [2]. This is important as efficient monitoring of neurological deterioration and understanding when to report novel or developing symptoms is critical [40]. The decision to undergo surgery is a significant consideration, as evidenced by the frequent queries of people with DCM about the suitability of surgery and the best type of surgery for their circumstances [13,37]. For those treated surgically, a range of surgical approaches can address the degenerative changes in the cervical spine [41]. Nonetheless, the decision to offer surgery and its timing is nuanced owing to its risks and the potential for DCM to remain mild [42]. Furthermore, surgery seldom leads to a full recovery; instead, a heterogeneous and lifelong disability is generally expected [8,10-13]. The implications of this can involve multiple distinct complex care pathways, necessitating collaboration across an array of health care disciplines less familiar with DCM [43]. Therefore, it is essential that people with DCM are well prepared to navigate a host of complex decisions. Finally, although treatment decisions are often the focus of SDM tools [44], they represent one of many care decisions that patients encounter [45]. Our experience at Myelopathy.org, a DCM scientific and clinical charity, is that patient health care inquiries encompass a broad spectrum, ranging from “should I modify my day-to-day activities?” to “should I undergo a dynamic MRI?” This variability underscores the diverse range of decisions that people with DCM confront, necessitating decisional support. It is also our experience that these inquiries are often hindered by patients’ inadequate knowledge and perhaps misconceptions of DCM [45]. In this regard, people with DCM are poorly supported by a lack of comprehensive, patient-centric, and up-to-date educational resources [45].

The aforementioned considerations, coupled with the inherent recurrent nature of decision-making in DCM, underscore the pressing need for people with DCM to engage in meaningful dialogue with health care professionals (HCPs) and have access to high-quality decisional support. In line with other chronic illness research, we hypothesize that a structured and well-orchestrated communication framework, as formulated within SDM tools, is ideally placed to successfully guide people with DCM through their health journey. Thus, investigating this area is warranted. In addition, the strategic incorporation of SDM has the potential to aptly support knowledge translation endeavors by identifying critical knowledge gaps and providing a framework for the assimilation of new knowledge into clinical practice [45-47]. This is currently a major challenge in DCM, where awareness of the condition continues to be suboptimal in the medical profession [48]. This is even conspicuous among spinal surgeons, who are considered authorities in the field of DCM [49,50]. To illustrate, a retrospective analysis of surgical decision-making in the United Kingdom found that only the amount of cord compression (as opposed to the guideline recommendations of disease severity) predicted the likelihood of a patient being offered surgery [49,51-53]. As the patient is perhaps the most motivated stakeholder and, importantly, a more recent student of DCM, addressing SDM may also support more consistent and immediate evidence-based care [54-57].

### The Rationale for Developing a Core Information Set and Process Mapping Patients’ Health Care Journeys

Patients’ understanding of their baseline circumstances represents a fundamental starting point for SDM [58]. This is crucial for patients to aptly recognize care options and articulate their preferences [59]. Core information sets (CISs) were pioneered as a means to support informed consent before surgery [60-62] and comprise a distilled list of information items akin to a checklist. Their purpose is to guide an educational conversation between HCPs and their patients by ensuring that critical information about a topic is shared and considered. CISs can help reduce variation in the practice of providing information to patients [63]. Their structured and condensed nature can also circumvent the potential for information overload [64]. Therefore, a DCM CIS centered on delivering information material to the diagnosis stage could assist the efficiency of clinical interactions and quality of onward health decisions by improving patients’ knowledge of the condition and their circumstances. As CISs are coproduced using multistakeholder input, their consensus-derived nature would also ensure that the information considered is of agreed importance to both professionals and patients.

Beyond diagnosis, the successful development of SDM tools depends on characterizing the critical decision points along patients’ care pathways [65]. A key aspect of quality improvement interventions is identifying whether and when a decision is required [66,67]. Process mapping, the diagrammatic representation of workflows, is frequently used in health settings to understand care processes [68]. By capturing all relevant steps in a sequence of actions, a process map can provide a comprehensive overview of an activity and its potential strengths, vulnerabilities, and limitations. As with CISs, process maps are coproduced via the input of key stakeholders who describe the workflows and cognitive decisions associated with a process [69,70]. Therefore, process mapping the key decisions of people with DCM is instrumental in paving the way for the development of SDM tools, ensuring their foundation in clearly delineated needs.

This research protocol describes the preliminary groundwork required to enable the exploration of SDM tools for DCM. First, we outline the development of a DCM CIS tailored to guide a material discussion between professionals and their patients at diagnosis. Second, we detail the procedure for formulating a global DCM process map that comprehensively captures common critical decisions in the patient journey.

## Methods

### Overview and Scope

#### Myelopathy.org and the Research Objectives and Common Data Elements for Degenerative Cervical Myelopathy Community

Myelopathy.org is the first and currently only scientific and clinical charity dedicated to improving the outcomes of people with DCM [71]. The charity has a global professional and lay audience, making it well suited for spearheading the initiative described in this protocol, which forms part of a larger enterprise aimed at promoting SDM in DCM. This will occur through the Research Objectives and Common Data Elements for Degenerative Cervical Myelopathy (RECODE-DCM) platform, an international community of professionals with diverse expertise in spinal care, and people with DCM registered with the charity working together to accelerate knowledge discovery in DCM.

The community was formed in collaboration with AO Spine (a nonprofit organization supporting education and research for spinal surgeons) to develop a research toolkit aimed at accelerating knowledge discovery in DCM [1,4,18,72]. Recognizing the broader opportunities for this community to address knowledge gaps in DCM and support their implementation led to its recognition as a distinct entity [73-75]. The network is now maintained by Myelopathy.org under its advisory board.

The research team formulated 2 objectives for this study, as outlined in the following sections.

#### Objective 1: To Enhance Knowledge of the Condition Among People With DCM

This will be achieved by developing the first DCM diagnosis CIS using an international multistakeholder (Delphi) consensus process.

#### Objective 2: To Identify Critical Decision Points in the Medical Care Product Life Cycle That May Benefit From SDM Tools

This will be achieved through a process map exercise involving an international multidisciplinary team of key stakeholders, including patient representatives.

Information on the status of each phase is shown in Table 1.

**Table 1:** Research study stages, findings summary, and status.

Description			Findings summary		Status
**Core information set**					
	Scoping literature review of educational resources in DCM^a^	>80% of resources geared toward professionals		Completed	
	Semistructured patient interviews	Varied information needs throughout the patient journey		Completed	
	Survey of professionals	Survey of professionals to collect their views on the information that people with DCM need		Completed	
	Delphi consensus survey of professionals and patients	Pending		In preparation	
**Process mapping**					
	Preparation, planning, and process identification	Pending		Completed	
	Data gathering	Pending		In planning	
	Process map generation	Pending		In planning	
	Analysis	Pending		In planning	
	Taking it forward	Pending		In planning	

^a^DCM: degenerative cervical myelopathy.

### Study Design

Enacting SDM requires insight into decisional dynamics. At its core, this entails discerning the critical timing of decisions and navigating factors related to decisional conflict—a state of uncertainty over a course of action when multiple options are present, each requiring careful evaluation of risks, benefits, and alignment with personal values. Key among the latter is an understanding of the informational demands associated with each decision-making juncture. To meet these requirements, our protocol proposes a mixed methods design that combines the structured, quantitative Delphi consensus procedure with the qualitative, visual approach of process mapping. By leveraging both methods, our study aims to translate evidence-based knowledge and stakeholder experiences into clinically actionable tools for enhancing the health outcomes of people with DCM [76]. The following sections elaborate on our application of the Delphi procedure in constructing the first DCM CIS and process mapping to highlight patients’ key decision points.

### Objective 1: To Enhance People With DCM’s Knowledge of the Condition—Developing the First CIS for Use in DCM at Diagnosis

#### Delphi Consensus Process Overview

**Figure 1:**
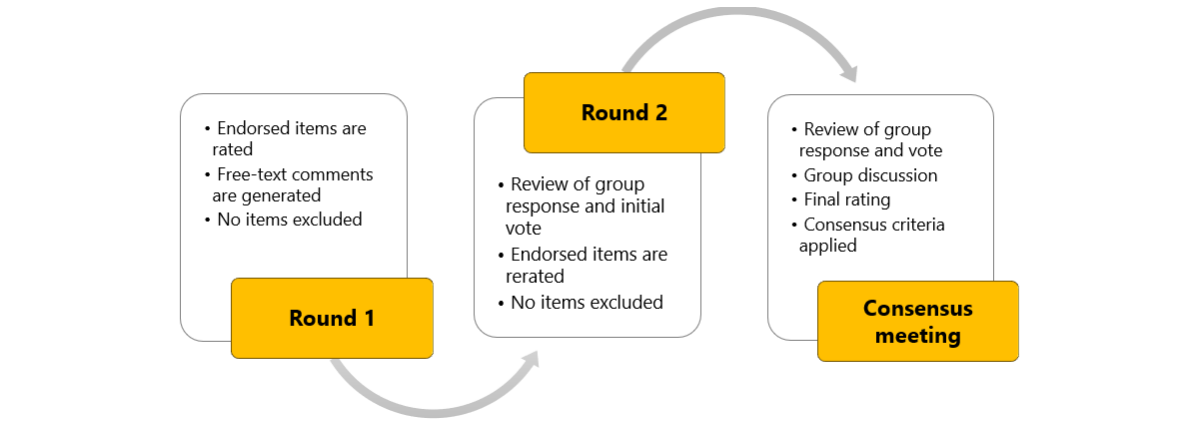
Overview of the Delphi rounds and consensus meeting.

The Delphi technique is a well-established method for achieving consensus among experts on a given topic [77]. It is particularly suited to contexts in which knowledge is ambiguous, empirical evidence is scarce, or priority setting is required [78]. In this procedure, a panel of preselected experts are engaged in a series of structured surveys. Each round incorporates controlled feedback, usually a blend of quantitative and qualitative data, the purpose of which is to foster insights from previous rounds, prompting reflection and the potential revision of viewpoints [79]. Designed to incrementally foster a convergence of opinions, the procedure is usually conducted anonymously, allowing participants to freely articulate their perspectives [80]. The number of rounds, execution of question delivery and response acquisition, and standards of achieving of “consensus” can vary within this general framework. Figure 1 provides a schematic overview of our approach, comprising 2 anonymized web-based surveys followed by a consensus meeting hosted over the internet. Consensus meetings offer a platform for real-time dialogue [81]. This face-to-face component has been observed to play a role in refining and enhancing the group’s collective understanding, paving the way for a more sophisticated consensus [82].

**Figure 2:**
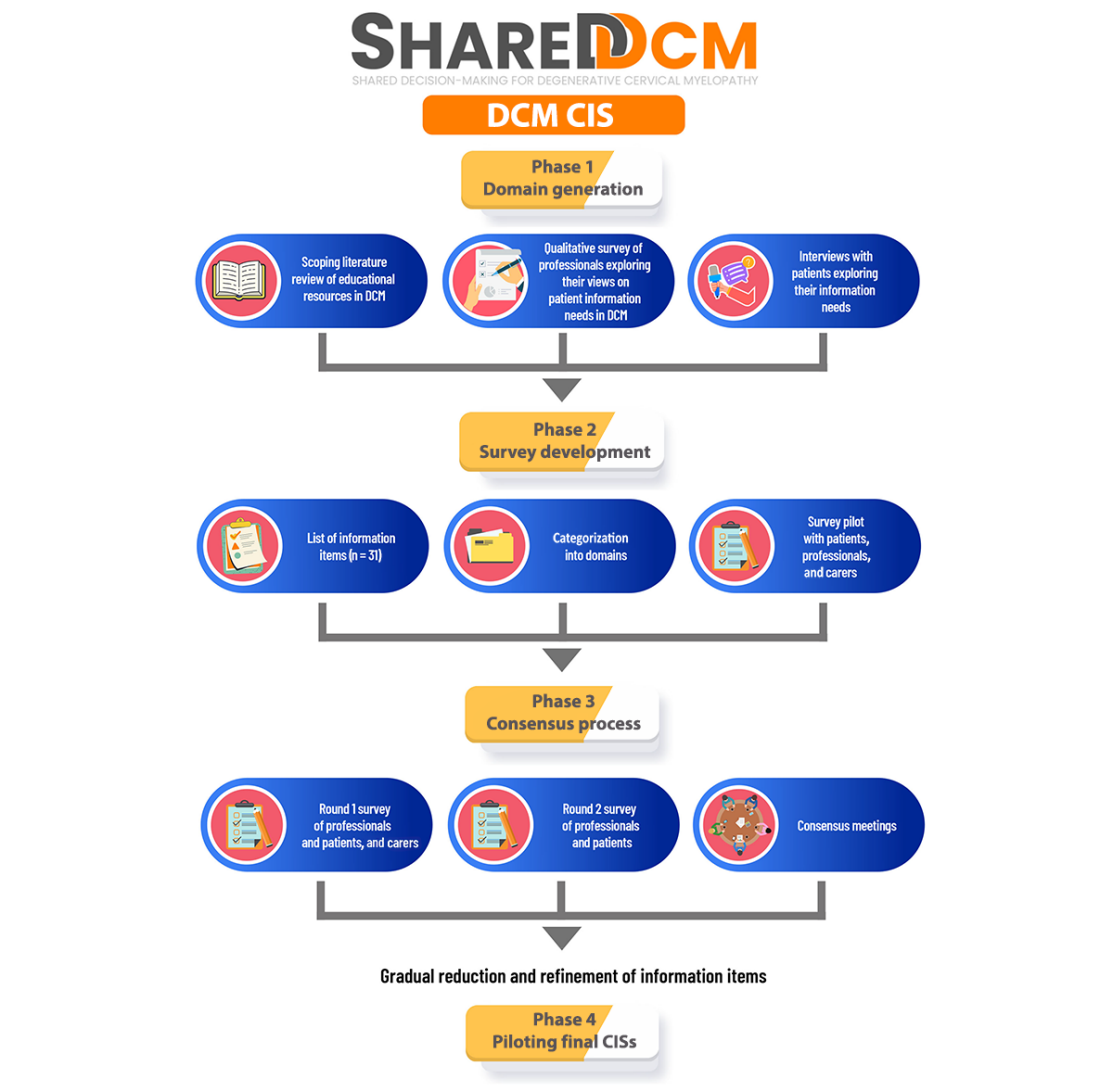
Overview of the phases involved in the development of the degenerative cervical myelopathy (DCM) diagnosis core information set (CIS).

The Delphi method has been widely used in health sciences [83] to generate clinical recommendations [84] and standards and guidelines for theoretical or methodological issues [83,85,86] for a range of chronic illnesses such as diabetes [87,88], cancer [89,90], asthma [91], rheumatoid arthritis [92], and spinal cord injury [93,94]. Our objective using a Delphi procedure is to develop a CIS that facilitates optimal communication of information to people with DCM about the condition at the point of diagnosis. Figure 2 shows an overview of the phases involved in developing the CIS.

#### Sample Size

The optimal participant head count for a Delphi study remains a subject of ongoing discussion [82], as exemplified by the historical variance in sample sizes [95]. However, representativeness is not shaped by conventional sampling strategies [96,97] but rather through the expertise of the panel members [98]. Guidelines suggest a minimum of 7 to 15 panelists for optimal reliability [78]. Some believe that exceeding 30 participants adds little value [99-101]. To develop a relevant CIS, our study aims to target the 2 primary DCM stakeholders: people with DCM and HCPs managing DCM. We propose enlisting at least 15 experts from each category, with no upper limit [78,95,102]. We address potential carer involvement in the following section.

#### Expert Selection

The sample composition significantly determines the validity of the study results [103,104]; therefore, an element of careful judgment is warranted to ensure a “balanced” panel [104-106]. To ensure a judicious selection of experts, this study will use a purposive sampling approach, drawing surgeons, other HCPs, and people with DCM as participants from the specialized RECODE-DCM community. People with DCM will be considered if they (1) have been formally diagnosed with DCM and are at least 6 months after surgical decompressive treatment, the time point at which neurological recovery is considered to halt for most [107], or (2) have undergone at least 6 months of nonoperative management [49]. Professional stakeholders will be invited to participate if they (1) are experienced in managing DCM for at least 5 years; (2) belong to key clinical disciplines, including spinal surgery, neurology, combined physical and psychological rehabilitation medicine, physiotherapy, pain management, and primary care [4,108]; and (3) have no conflict of interest in developing a CIS. All participants will be English-speaking adults (aged ≥18 years).

A diverse and geographically representative panel will be sought, in keeping with the aim of developing an internationally suitable CIS. Adequate patient involvement is vital to this endeavor to help foster evidence-based decision-making [109]. Recognizing the importance of patient perspectives, we propose aiming for a stakeholder target ratio of 1:1:2 (surgeons:other HCPs:people with DCM). The increased representation of people with DCM addresses their typically minor role in such studies and considers recent findings indicating a divergence in clinician- and patient-prioritized outcomes [4] and information needs [45]. This ratio also aligns with the status of people with DCM as the principal beneficiaries of the CIS and enhances its applicability from a patient and public perspective.

For balanced geographical stakeholder representation, a 1:1:1 target ratio is proposed, spanning North America and Australasia; Europe and the United Kingdom; and “elsewhere,” comprising Africa and Asia. Including stakeholders from these regions will help ensure that the CIS considers a wide range of health care systems, cultural practices, and resource availability. However, it is worth noting that engaging participants from the “elsewhere” category may prove difficult. These regions, which are often underrepresented in the research community owing to fewer research resources and infrastructure [110], are similarly underrepresented in our specialized RECODE-DCM community. Recognizing this potential recruitment challenge upfront, we will aim to achieve equal regional representation during the final consensus meeting, with no items removed before this stage.

Carers (informal and formal) of people with DCM will also be invited to participate in the Delphi process as they offer significant practical and emotional support to people with DCM [16] and likely hold unique perspectives with regard to their information needs at the time of diagnosis. Consultation with informal carers, often subject to significant caregiving strain [16], may indirectly contribute to their welfare by promoting improved standards of care. However, we acknowledge that the recruitment of carers may be challenging as they are less likely to self-identify, as documented in related research domains [111]. In addition, the number of carers involved in the RECODE-DCM community is currently limited. For these reasons, no stakeholder or geographical target ratios will be specified for this group. Their involvement will be encouraged by existing HCPs and people with DCM in the community.

Participants will be invited to complete round 2 of the Delphi survey only if they have responded to round 1. A dropout rate of approximately 20% is expected from previous Delphi exercises [95,112]. Respondents who complete the Delphi process will be acknowledged in published material. No monetary incentives will be offered.

#### Generating Information Items

The first step in the Delphi process involves generating a list of information items for the voting consensus process. The procedure comprises 3 steps, described in the following sections.

##### Scoping Literature Review and Patient Interviews

In structuring the Delphi procedure, we sought to cover the wide spectrum of DCM-related information with the potential to foster patient understanding of the condition, facilitate informed decision-making, and promote efficient self-management. Clinically pertinent items were derived from scientific publications, patient leaflets, health websites, and videos identified in a scoping review of DCM education resources [38]. In total, 150 information resources were reviewed, spanning 115 (76.7%) scientific publications, 28 (18.7%) videos, and 7 (4.7%) health organization resources and health education websites. Items sourced in this manner characterized the disease etiology, pathophysiology, epidemiology, management, and outcome prediction. Additional multidimensionality was added via items sourced from qualitative research involving patient focus groups [10,113] and semistructured interviews [45]. These provided valuable additions focused on the impact of DCM on employment, mental health, and quality of life. The process of deriving the information items was iterative. Each new unique piece of information was noted as an item, and multiple returns to the data were made to ensure the avoidance of duplicates and omissions. This process continued until a preliminary list of information items (n=95) was collated and no new items could be identified.

##### Professionals’ Perspectives

Professionals’ views on the information items to be included in the diagnosis CIS were identified using an open-ended qualitative questionnaire (SurveyMonkey; Momentive Inc). Respondents were provided with relevant information via a web hyperlink to the Participant Information Sheet. The sheet included a summary of the research project, its objectives, the inclusion and exclusion criteria, anonymity and confidentiality, and the anticipated time commitment required to complete the survey. To stimulate participant engagement, a short educational video explaining the purpose of the procedure was presented [114]. Biographical information, including training specialty (surgery or other HCP), was collected from consenting participants. Respondents were subsequently directed to a page containing the following open-ended question: “What information do you think people with DCM need to be told at the point of diagnosis about their condition?” It is acknowledged that free-text questions do not generate rich qualitative data [115]. However, our objective was not to elicit robust qualitative insights but rather to generate a list of information items from the professionals’ perspectives. To mitigate this limitation, participants were asked to provide a short rationale for each recommended information item. This approach can act as a proxy for qualitative interviews administered to convenience samples [116] and allowed the study team to improve evaluative rigor by understanding the rationale behind the recommendations received. There was no limit on the number of information items that the HCPs could submit.

To facilitate reflective consideration from respondents, participants were presented with a word cloud upon submitting their initial recommendations (Multimedia Appendix 1). Word clouds are a graphic representation of textual data that use frequency counts to display effective summaries of common themes within a given text [117] and have been successfully used previously in DCM consensus work [118,119]. The word cloud generated for this study was produced using qualitative codes derived from the thematic analysis of interviews with people with DCM on the topic of their information needs [45]. This approach was selected to mitigate issues related to language variability that can arise from using direct quotes from the participants, which may be influenced by individual differences in language proficiency, education, and cultural background, and to improve the comprehensibility of the word cloud. Similar adjusted approaches have been used by others [117]. Participants were asked to reconsider their recommendations after viewing the word cloud. As word clouds have been found to promote critical thinking and reflection [120], we hypothesized that this approach would help stimulate professional stakeholders to generate insightful recommendations that are better aligned with patients’ needs. The open-ended questionnaire was not issued to people with DCM as their perspectives have already been collected [45].

##### The Questionnaire

A total of 46 respondents, predominantly spinal surgeons (n=33, 72%), suggested information items (n=312) for inclusion in the Delphi survey. Contributors also included physiotherapists (8/46, 17%) and other HCPs, including neurologists, family physicians, allied health professionals, and researchers (5/46, 11%). The items received were carefully scrutinized for redundancy. On inspection, suggestions paralleled our existing list, demonstrating a broad alignment across all information sources, including the expert clinician group. A multidisciplinary study committee comprising 3 people with DCM, 3 neurosurgeons, a neurologist, 2 consultant physiotherapists, a lay representative, and the study coresearcher condensed the list to 31 information items and classified them into 8 distinct categories deemed to accurately represent their content. For the purposes of developing a CIS, the term “information item” was conceptualized as overarching categories or topics that house more specific, detailed information. For example, the item “lifestyle factors” was an inclusive banner for detailed subjects such as the effects of smoking and occupational trauma on DCM. This approach was chosen to ensure the appraisal of *meaningful* concepts or core educational aspects. The detailed content within each item, although not voted upon, will be accessible for participants’ reference throughout the Delphi study. Categorizing information items in Delphi studies is crucial in reducing redundancy and ensuring that all relevant concepts are captured as well as promoting consensus building among expert panelists by clarifying the meaning of each item [79,80]. This also facilitates a systematic and standardized approach to synthesizing diverse perspectives, thereby generating informed recommendations [79,121]. In preparation for pilot-testing, the research team created a draft survey based on the final list and evaluated the software settings, automation, flow, ease of navigation, and completion time. For ease of navigation, only 1 information item was presented per page [122].

#### Pilot Survey

Pilot-testing was conducted with the study committee. Feedback concerning the language, structure, readability, comprehension of statements, and formatting of the questionnaire was collected through open- and closed-ended questions.

#### Delphi Survey

An email campaign will be created in Gmail (Google) using a newsletter layout template and sent to HCPs, people with DCM, and carers in the contact directory of the RECODE-DCM network using the multisend function. The multisend option is a built-in mass-email feature that allows emails to be sent in bulk to distinct recipients, thus preserving their anonymity, which is a key feature of the Delphi method [98]. Participants will have the option to opt out of the process at any point through a unique “unsubscribe” link automatically added to each email. Recipients who unsubscribe will be removed from future email campaigns. The email will provide prospective Delphi panelists with a brief overview of the study. Potential panelists will be directed to Delphi round 1 via a SurveyMonkey web link embedded in a participation button at the bottom of the email. The survey will open to a short explanatory video and a web link to the Participant Information Sheet, which will provide a summary of the research project, aims, the inclusion and exclusion criteria, anonymity and confidentiality, and anticipated time commitment. The following biographical data will be collected for people with DCM: age, year of diagnosis, history of surgery or nonoperative management, and employment status. For HCPs, information about their profession, specialty, job title, and experience in managing DCM will be captured.

Panelists will be asked to independently rate the importance of information items using a 4-point Likert scale (“essential,” “very important,” “unimportant,” and “not at all important”). This range of response categories is more likely to produce stable findings in Delphi studies [95]. Respondents will also be asked to provide a brief rationale for their responses and whether the information items presented could be modified to improve their relevance using a comment box. It is common practice to include in questionnaires that respondents themselves complete an invitation to add issues not covered in the main part of the questionnaire or to expand on terms [123]. The presentation of information items will be randomized using a pseudorandom number generator throughout both rounds [124]. No more than 3 rounds will take place to ensure that the process does not become too repetitive and time-consuming to maintain an adequate response rate [80]. After each round, participants will be asked to read the feedback before responding again to statements.

#### Data Analysis

##### Overview

Qualitative and quantitative summaries will be used to understand areas of agreement or disagreement between panelists, generate feedback, and stimulate participants’ reflections. All forthcoming data analyses will be carried out by a postgraduate research assistant. Both quantitative and qualitative methods will be used to analyze interround feedback, and the findings will be reported to the panelists and the core research team after each round. The provision of both types of data is essential to enhance rating accuracy [125].

##### Qualitative Analysis

Free-text comments will be analyzed using inductive content analysis [126], a procedure commonly used with text-based data. Its pragmatic emphasis on theoretical constructs makes this approach particularly suited to practically oriented research where the goal is to describe a phenomenon of interest [127] and generate answers for the application of findings [128]. A summary of the remarks made by other panelists will be displayed anonymously to participants after each round.

##### Descriptive Statistics

Following each round, panelists will receive aggregate-level summary statistics for each item in the form of frequency percentages, medians, ranges, and IQRs [129]. Participation and attrition rates will be summarized as frequencies and percentages after round 2. Participants will be asked to read the summarized feedback before responding to round 2 statements.

##### Interrater Stability

Descriptive statistics reflect the level of overall consensus in a given sample but may not account for random change agreement and, therefore, may fail to accurately depict “true” consensus [130]. The κ statistic measures agreement beyond that expected by chance [130]. A weighted κ coefficient will be calculated after round 2 for the between-participant agreement on each information item. This variant is preferred compared with its unweighted equivalent as it is more suited to ordinal data given its ability to account for the magnitude of differences between the agreement levels [131]. The κ statistic will be particularly helpful for guiding the direction of conversations during the consensus meeting, ensuring that discussions are rooted in objective findings. κ calculations will be provided for each information item along with the reference value description of the agreement level in accordance with the study by McHugh [132] (Table 2).

No information items will be removed during these rounds. Data analyses will be conducted using SPSS (version 26; IBM Corp), and graphs will be produced using Microsoft Excel (Microsoft Corp).

**Table 2:** Level of agreement represented by κ values^a^.

κ value	Agreement level
0.0-0.2	None
0.21-0.39	Minimal
0.40-0.59	Weak
0.60-0.79	Moderate
0.80-0.90	Strong
≥0.90	Almost perfect

^a^Adapted from the study by McHugh [132].

##### Consensus Criteria

Delphi studies lack consensus on the specific threshold for agreement. Significantly, Linstone and Turoff [133] posit that an agreement of >80% is neither the central objective of the Delphi process nor always practically possible. In this study, items rated as “essential” or “very important” by ≥75% will qualify for inclusion, whereas those judged as “unimportant” or “not at all important” by ≥75% will qualify for exclusion. Items not fulfilling these criteria will be defined as not having reached a consensus. This threshold value [134,135] is situated between the 50% consensus supported by some [136,137] and the 100% cutoff point suggested by others [138]. Consensus criteria will be applied after the consensus meeting.

To gain additional insights into the perspectives of the 3 stakeholder groups, comparative statistics will be calculated after each round for review by the core study team. The parametric 1-way ANOVA will be used to investigate differences in stakeholder responses during each round if the assumptions of normal distribution and homoscedasticity are met [139]. These will be inspected using histograms and the Shapiro-Wilk test and the Levene test for equality of variances, respectively. Statistically significant results will be investigated using post hoc analyses to be chosen in accordance with whether homoscedasticity is met [140]. In the event of violations of the assumptions of normality, the Kruskal-Wallis *H* test, a nonparametric alternative, will be used as it is predicated on rank-based assessment and does not presuppose normality or homogeneity of variance [141]. Moreover, in the event that we are unable to identify sufficient carers to complete the Delphi survey, we will use the independent-sample 2-tailed *t* test to investigate statistically significant differences between HCPs and people with DCM. If the assumptions for the test are not met, we will use its nonparametric equivalent, the Mann-Whitney *U* test. Significance will be assumed at *P*<.05.

#### Consensus Meeting

The consensus meeting via Google Meet (Google LLC) will be attended by a maximum of 20 randomly selected HCPs, people with DCM, and carers who have completed both rounds of the Delphi survey, preserving the key stakeholder and geographical representation previously described. Participants will be presented with round 2 voting results, as previously described, and asked to anonymously rate the importance of each item for a final time using the polling tool. Frequency percentages will be generated in real time and presented to the voters on-screen to facilitate discussion. The facilitator will guide further discussion regarding any items garnering equal consensus (50%) between the agreement (“essential” and “very important”) and disagreement (“unimportant” and “not at all important”) before asking participants to vote again. In all other cases, items failing to meet the predetermined ≥75% consensus score will be eliminated.

#### Reporting the Survey Results

To ensure a thorough description of the Delphi process, survey results will be reported following the Checklist for Reporting Results of Internet E-Surveys [142].

### Objective 2: To Identify Critical Decision Points in the Care of People With DCM—Process Mapping the Patient Journey

#### Overview

Critical decision points in the trajectory of patients will be identified using process mapping, a technique that can aptly describe the workflow and cognitive procedures associated with an activity at the desired level of granularity [143]. We define a critical decision point as a distinct juncture in the care pathway when a professional-patient discussion leads to a care decision. These are essential “go-or-no-go” decisions [59]. Our process map protocol follows the quality criteria framework set out by Antonacci et al [69] to ensure compliance with best practice recommendations. Our approach to each phase is described in the following sections.

#### Preparation, Planning, and Process Identification

The process map exercise described in this protocol is guided by the need to characterize common critical decision points that adequately represent the global DCM health care pathway. To obtain broad insights, we define the starting point as the time when a person with DCM is diagnosed with DCM and the end point as the time when a person with DCM engages in posttreatment management of the condition. We will define the patient group under investigation as people with moderate or severe DCM treated surgically and people with mild DCM treated nonoperatively [2]. This approach will ensure an all-encompassing investigation of the care route for people with DCM.

#### Steering Group

It is good practice for process map exercises to be led by a steering group comprising at least one member with lived experience of the condition under investigation and a member with previous process map experience [144]. Following these recommendations, our process map activities will be supervised by the same multidisciplinary study committee overseeing the Delphi consensus process. At a minimum, the committee is envisaged to meet at the beginning and end of project activities, with further meetings as required. Decisions made at meetings will be binding when the meetings are quorate, that is, attended by at least one member of the core research team, one patient representative, and one member with process map experience. Project-relevant communications may also take place via email correspondence as necessary.

#### Recruitment Strategy and Skills Training

Key informants—English-speaking adults (aged ≥18 years)—will be drawn from the specialized RECODE-DCM community using a convenience sampling approach. Professionals will be approached for participation if they belong to key clinical disciplines, including spinal surgery, neurology, combined physical and psychological rehabilitation medicine, physiotherapy, pain management, and primary care [4,108], and have at least 5 years of experience in managing DCM. People with DCM will be eligible if they have undergone surgical decompressive treatment at least 6 months before, the time point at which neurological recovery is considered to halt [107], and if they have experienced the common care pathway for spinal disorders from primary care to rehabilitative services [145]. This criterion will ensure that participants with DCM are familiar with a wide range of interventions and services along the global spinal care pathway and, therefore, can provide an informed view of its workflow and cognitive decision points. To ensure a global representation of the spinal care workflows and cognitive decisions, both professionals and participants with DCM will be sought at the previously described geographical target ratio. In addition, a 1:1 ratio corresponding to HCPs to people with DCM will be sought in the development of the baseline process map.

An electronic study pack will be provided to those interested in participating, comprising a written study summary, this study protocol, and a Participant Information Sheet containing the study investigators’ contact details. To equip respondents with the necessary process map skills, all consenting participants will be issued a short explanatory document outlining relevant process map symbols alongside an example of a finalized map from a different health care process. Participants will be given the opportunity to contact the study team should they have any questions or require further clarification before commencing study activities.

#### Data Gathering

To enhance accessibility and foster participant engagement [146], data will be collected using one-to-one semistructured interviews, estimated at approximately 60 minutes and conducted via Google Meet. The interview schedules will be grounded in the literature, aiming to capture information on each step of routine clinical care under investigation [144,145]. To enhance comprehensiveness and incorporate expert input, the topic guide will undergo review and approval by the aforementioned steering group. In total, 2 distinct interview guides will be developed to interview clinicians and people with DCM. For clinicians, we propose a tailored approach in recognition of the specialization inherent in each HCP’s role. For example, primary care physicians are instrumental in recognizing the condition and its associated clinical examinations for referral to a spinal surgeon [42]. Specialist surgeons, under the remit of neurosurgery or orthopedics, play a pivotal role in steering the course of management [147]. Specialist practitioners such as physiotherapists play a vital role in assisting in the management of DCM via preoperative physiotherapy and neurorehabilitation programs, primarily used in the context of DCM to manage disability postoperatively [40,148]. Therefore, our interview guide will account for differences in specialists’ knowledge of care for DCM by asking HCPs an open question regarding how they manage people with DCM in their role specifically. From there, prompts relevant to each clinician’s profession and based on the information they provide will be used to guide the interview. By grounding the topic schedule in the particularities of each professional’s role, our approach can help ensure a thorough exploration of clinicians’ perspectives, accounting for the nuanced differences across specialties and individual clinical roles within the DCM care pathway. We also recognize that the care pathways of people with DCM differ owing to variations in patients’ DCM, flow times between different regions in the world, and guidelines of care [149]. Therefore, patients will be encouraged to share their experiences navigating the care system, focusing particularly on key decision-making points. Prompts will be used to elicit detailed accounts of their journey, the information sources they relied on, their understanding of their condition at each stage, and their level of satisfaction with the decision-making process. Interviews will be conducted until data saturation is reached, that is, until no new information is identified with respect to the research question [150]. At a minimum, it is envisaged that this will involve 20 interviews with HCPs and people with DCM. To allow for the incorporation of new insights generated during the data collection process, the interview guide may be adapted as necessary [151].

A baseline map will be developed by interviewing 3 surgeons and 3 people with DCM from each of the geographical target areas. This approach seeks to ensure that international perspectives are adequately represented from the onset. As surgical intervention is the cornerstone treatment for DCM and surgeons preponderate the research community [50], professional transcripts derived from surgeons specifically (rather than other HCPs) will inform the preliminary map. As women remain underrepresented in clinical surgical practice [152], we will seek to recruit the views of at least one female spinal or orthopedic surgeon in the development of the baseline map. This preliminary map will be developed iteratively through a series of additional interviews with surgeons, other HCPs, and people with DCM while ensuring geographical representation. Interviews will be audio recorded and written informed consent will be sought from all participants. Following their interview, each participant will be provided with a copy of their transcript, a process map created from their interview data, and the evolving baseline map. Participants will be asked to reflect on their interviews and consider whether they wish to supplement the process map. This approach will be used as a means of ensuring data validation and quality and promoting further unbiased contributions to the data [153,154].

Upon reaching data saturation, a selection of interviews will be chosen based on their richness, and the final process maps will be refined by referring to them. Data richness will be defined as “thick” data that best capture the multifaceted complexities of the process under investigation [155]. Participants will be assigned unique study numbers to anonymize their identities in the transcripts.

#### Map Generation

The baseline map will be developed and schematically represented using Lucidchart (Lucid Software Inc), a web-based diagramming application, and developed iteratively based on information obtained during subsequent interviews. A level of complexity associated with ad hoc variation in the international processes and cognitive decision points is anticipated. To accurately depict this, several process maps may need to be generated. For example, a separate process map may need to be created for preoperative, operative, and postoperative processes. Where possible, relevant clinical pathway documents provided by participants will be used in the development of the process map.

#### Data Analysis

Interviews will be transcribed verbatim and analyzed using inductive thematic analysis according to Braun and Clarke [156]. This will involve a coding process whereby segments of text are systematically organized according to common concepts and broader themes developed to encapsulate the main topics present in the interview data. The synthesis of these themes will be used to develop a visual representation of the interview data within the process map. The map will be developed iteratively, ensuring the inclusion of emerging themes. The final stage will involve validation to ensure the accuracy and reliability of the process map. This can be achieved through methods such as member checking, peer debriefing, or triangulation, ensuring the credibility and dependability of the findings [157]. The final process maps will be shared with the key participating stakeholders for comments and validation over email. Participant demographic characteristics will be collected during the interviews and presented in tabular form using frequencies, means, and SDs as appropriate. For HCPs, demographic data will include age, gender, job title, a brief description of their duties, and number of years managing DCM. For people with DCM, this will include age, gender, occupation, year of diagnosis, and treatment (where applicable).

#### Future Steps

Process maps should be used to guide process improvement initiatives [158]. The process maps generated following this study protocol will inform future efforts by Myelopathy.org to develop SDM tools that will help people with DCM participate in making specific and deliberate choices regarding their health. In particular, at the time of writing this protocol, it is considered that identifying critical decision-making points will allow for the development of widely used evidence-based SDM tools such as patient decision aids [159-162] or patient road maps [28].

### Ethics Approval

The University of Cambridge Human Biology Research Ethics Committee has approved the study (approval HBREC.2023.04).

### Informed Consent, Data Protection, and Dissemination Strategies

Written informed consent will be sought from participants for both study objectives, that is, in the development of the CIS and process map exercise. All data will be handled under UK data protection regulations. Dissemination strategies for this project will include scientific publication and communication of findings via the Myelopathy.org website and Myelopathy Matters podcast.

## Results

Recruitment for the Delphi consensus study began in April 2023. The pilot-testing of process map interview participants started simultaneously, with the formulation of an initial baseline map developed at the end of June 2023.

## Discussion

The proposed study protocol outlines a Delphi consensus process that draws on the expertise of clinicians experienced in managing DCM and of people with DCM to formulate a diagnosis-specific CIS. It provides the rationale for undertaking an international process map exercise to characterize the common decision points in the health journey of people with DCM and outlines a strategy for generating outputs that aptly fit the needs of the global DCM community via broad stakeholder consultation.

### Significance of the Study

To the best of our knowledge, this is the first initiative that aims to engage people with DCM in the management of their care. Given the high global incidence of DCM [2,163], the outputs of this preliminary study are relevant to a substantial cohort of people with DCM, clinicians, and policy makers worldwide. Most importantly, the CIS aims to address the significant educational deficit identified in DCM [38,45]. Drawing on the tangible impact of structured patient education [164-169], we anticipate that the CIS has the potential to reduce knowledge discrepancies and advance equitable opportunities for quality health management decisions. In particular, as CISs are developed using key stakeholder input, they are ideally placed to facilitate a well-orchestrated communication that mitigates the biases or inadequacies associated with purely professional-led communication [60,63]. Furthermore, educating patients on the condition is one facet of improving their openness to and, arguably, time to surgery [45], potentially driving improvements in health care outcomes [170,171]. This is important as many people with DCM misunderstand the necessity and immediacy of surgery [45]. An appraisal of the CIS will be undertaken following its implementation to assess its efficacy in clinical practice.

In addition, we anticipate that the benefits of the process map in the international DCM care pathways may extend beyond SDM, for example, to addressing timely treatment and improving knowledge exchange [69]. Characterizing the key decision points and matching them to the current evidence base may expose critical knowledge gaps that must be served with targeted research. This means that, as the evidence is updated, so can be the SDM tool, helping accelerate the adoption of evidence into practice. In addition, providing a tangible visual tool to convey the patient experience across different health care settings and among various stakeholders may enhance communication among multidisciplinary teams [69], a recognized objective in DCM [172]. In the long run, the process map can serve as a valuable tool for patient advocacy, spotlighting areas of the care process that are particularly challenging [45], allowing policy makers and care providers to be better informed about the realities of living with DCM. This takes on added significance in low- and middle-income countries [173], where the burden of DCM is high yet access to care remains substandard [149]. Finally, developing a globally shared understanding of DCM care, with its similarities and differences, may facilitate opportunities for local redesign, development, or evaluation [69], leading to improvements in access to treatment [13].

### Strengths and Limitations

The proposed study has several methodological strengths and limitations. First, the Delphi method offers an unbiased manner of soliciting consensus when this is lacking [174]. The guarantee of anonymity reduces expert-layperson power asymmetries between clinicians and patients, which are inherent in typical health care interactions [175]. This provision is particularly important to people with DCM, the beneficiaries of the CIS, to assert their viewpoints without reservation. Furthermore, the provision of systematic and controlled feedback fosters transparency, dialogue, learning, and sometimes novel insights that might not have been apparent in isolation [78]. Through its systematic progression, the Delphi method allows for gradual refinement of ideas [176]. Information items put forward for consensus in this manner are likely to become more patient-centric, relevant, and clear, ultimately resulting in the production of a more pertinent CIS. In addition, conducting the survey over the internet broadens the inclusion of people with DCM, including presurgical DCM, which is poorly represented in the conventional literature [50,177].

This approach also has a number of limitations. Despite comprehensive efforts to source a broad range of expert participants, the conclusions of the study may suffer from limitations associated with the panel’s composition. This is the case with any Delphi study [178]. In this regard, our purposive sampling approach may lead to outputs that may not fully reflect all health care contexts and stakeholders’ perspectives. Pilot-testing the survey with a smaller representative sample can help ensure its comprehensiveness [179]. Expert judgment may also be influenced by cognitive biases, which is a well-recognized limitation of Delphi studies [79]. To mitigate this, we are curating an expert panel diverse in both roles and geographic locations to promote a more balanced collective judgment [180]. Other efforts to minimize bias throughout the Delphi process include the randomization of survey statements to eliminate primacy effects, requests to participants to include a rationale for their choices, and providing feedback in the form of median rather than mean values [116,124,125]. Compared with evidence-based approaches, expert surveys do not capture cause-effect relationships and are, therefore, definitive [181]. That is, our experts’ agreement on the final CIS does not assure the tool’s efficacy. To address this concern, the CIS will be evaluated, the specifics of which will be laid out in a separate study protocol.

Finally, inductive thematic analysis provides an adaptable methodology for developing the process map. Its flexibility allows for the generation of unanticipated themes and patterns [156]. Coding and organizing the data around common concepts aids in translating potentially complex data into a digestible visual representation [182]. The iterative nature of this method allows for continuous refinement of the process map, thereby increasing the reliability of the results [183], whereas validation strategies such as member checking and peer debriefing ensure the accuracy, credibility, and dependability of the findings [157].

### Conclusions

This is the first study to establish an international-led collaborative framework aimed at enabling SDM in the management of a chronic illness. To the best of our knowledge, this approach has not been used before. The projected benefits of this initiative are poised to improve people with DCM’s knowledge about their condition. In the long term, this study is intended to drive the development and deployment of SDM tools in DCM care.

## Appendix

Multimedia Appendix 1 Word cloud of qualitative codes generated from the thematic analysis of interviews with people with degenerative cervical myelopathy.

## Abbreviations

CIS: core information set
DCM: degenerative cervical myelopathy
HCP: health care professional
RECODE-DCM: Research Objectives and Common Data Elements for Degenerative Cervical Myelopathy
SDM: shared decision-making

## Data Availability

The data sets being generated and analyzed in this ongoing study are not publicly available at this time because of the need to preserve the integrity of the ongoing Delphi consensus process but will be shared in subsequent publications after the study concludes.
