# Supplementary figures and images for: Toward Shared Decision-Making in Degenerative Cervical Myelopathy: Protocol for a Mixed Methods Study
Source: JMIR Res Protoc. 2023 Oct 9;12:e46809. doi: 10.2196/46809 (PMC10594151; doi:10.2196/46809)

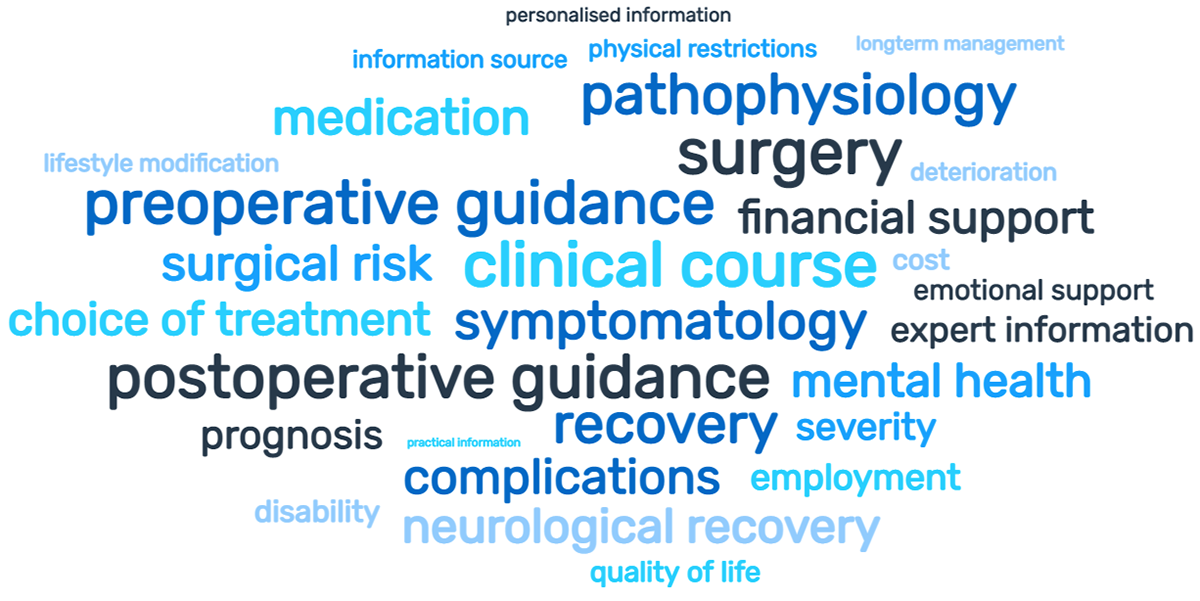

Supplement: Multimedia Appendix 1 [file resprot_v12i1e46809_app1.png]
